# Biological Activities of Phenolic Compounds of Extra Virgin Olive Oil

**DOI:** 10.3390/antiox3010001

**Published:** 2013-12-20

**Authors:** Maurizio Servili, Beatrice Sordini, Sonia Esposto, Stefania Urbani, Gianluca Veneziani, Ilona Di Maio, Roberto Selvaggini, Agnese Taticchi

**Affiliations:** Dipartimento di Scienze Economico-Estimative e degli Alimenti, Sezione di Tecnologie e Biotecnologie degli Alimenti, Università degli Studi di Perugia, Via S. Costanzo, Perugia 06126, Italy; E-Mails: b.sordini@gmail.com (B.S.); sonia.esposto@unipg.it (S.E.); stefania.urbani@libero.it (S.U.); gianluca.veneziani@progetti.unipg.it (G.V.); ilonadimaio@libero.it (I.M.); roberto.selvaggini@unipg.it (R.S.); agnese.taticchi@unipg.it (A.T.)

**Keywords:** Extra Virgin Olive Oil, phenols, antioxidants, healthy, sensory

## Abstract

Over the last few decades, multiple biological properties, providing antioxidant, anti-inflammatory, chemopreventive and anti-cancer benefits, as well as the characteristic pungent and bitter taste, have been attributed to Extra Virgin Olive Oil (EVOO) phenols. In particular, growing efforts have been devoted to the study of the antioxidants of EVOO, due to their importance from health, biological and sensory points of view. Hydrophilic and lipophilic phenols represent the main antioxidants of EVOO, and they include a large variety of compounds. Among them, the most concentrated phenols are lignans and secoiridoids, with the latter found exclusively in the Oleaceae family, of which the drupe is the only edible fruit. In recent years, therefore, we have tackled the study of the main properties of phenols, including the relationships between their biological activity and the related chemical structure. This review, in fact, focuses on the phenolic compounds of EVOO, and, in particular, on their biological properties, sensory aspects and antioxidant capacity, with a particular emphasis on the extension of the product shelf-life.

## 1. Introduction

Several epidemiological studies have demonstrated that adherence to the Mediterranean diet can be associated with longevity and with a reduced risk of morbidity and mortality [1,2]. The traditional Mediterranean food pattern encompasses a number of dietary components, which are thought to be associated with protective health effects within this nutritional pattern. In this context, Extra Virgin Olive Oil (EVOO) plays an important role as the main source of fats in the diet of this area (Reg EU 432/2012) [3]. EVOO is the fresh olive juice obtained exclusively by mechanical and physical processes. It consists of a major fraction of mono- and poly-unsaturated fatty acids (mainly triacylglycerides) representing more than 98% of the total weight, whereas the minor fraction (approximately 2% of the weight) is composed of a complex set of minor compounds, which includes over 230 chemical compounds (aliphatic and triterpenic alcohols, sterols, hydrocarbons, volatile compounds and antioxidants). Traditionally, the nutritional values of EVOO are due to the high monounsaturated fatty acid (MUFA) content, principally made of oleic acid. This represents the most abundant, monoenoic, fatty acid in olive oil, the concentration of which ranges from 56% to 84% of the total fraction of fatty acids. On the other hand, the linoleic acid concentration (which represents the major, essential, fatty acid and the most abundant, polyunsaturated acid in the Mediterranean diet) ranges between 3% and 21%. Several studies have suggested that MUFAs are effective in reducing LDL-cholesterol activity, even though the associated mechanisms are not well known.

Several studies carried out recently have demonstrated that the healthy effects should also be attributed to the Olive Phenols (OP) of EVOO [4,5,6,7]. According to EFSA (2011), the chemical composition of EVOO and its phenolic fraction in particular is effective in decreasing the risk of cardiovascular disease [3]. Moreover, several studies on EVOO [8] support the fact that the daily consumption of OP, estimated at 5 mg/day, has very important, healthy effects on humans because of the reduction in the peroxidation of blood lipids due to phenols. The main antioxidants in EVOO are represented by lipophilic and hydrophilic phenols [9], with the presence of a small amount of carotenoids. Moreover, lipophilic phenols (especially tocopherols and tocotrienols) can also be found in other vegetable oils. To this regard, over 90% of tocopherols in EVOO is made by α-tocopherol, the concentration of which is also characterized by a strong variation depending on pedoclimatic factors and agronomic practices, such as the area of origin, the cultivar and the stage of fruit ripening [10,11]. The data obtained assessing 430 samples of EVOO have showed a range of variability included between 23 and 751.1 mg/kg.

EVOO hydrophilic phenols represent a class of secondary plant metabolites demonstrating unusual sensory and health properties. Generally speaking, unlike lipophilic phenols, they are not found in other oils and fats [9,12]. The chemical composition of the hydrophilic phenolic fraction of EVOO has been studied extensively in the past. Different groups of phenolic compounds can be found in these olive oils, such as phenolic acids, phenolic alcohols, hydroxy-isochromans, flavonoids, lignans and secoiridoids (Table 1) [4,13,14]. The first groups of phenols discovered in EVOO were phenolic acids, including caffeic, vanillic, syringic, *p*-coumaric, *o*-coumaric, protocatechuic, sinapic, *p*-hydroxybenzoic and gallic acid [4,15,16,17]. In the past, phenolic alcohols, principally represented by (3,4-dihydroxyphenyl)ethanol (3,4-DHPEA) and (*p*-hydroxyphenyl)ethanol (*p*-HPEA), were discovered in EVOO. According to Montedoro *et al*. [16], their concentration is generally low in freshly pressed oils, but this increases during oil storage, as a consequence of the hydrolysis of EVOO secoiridoids, such as 3,4-DHPEA-EDA, *p*-HPEA-EDA and 3,4-DHPEA-EA, all containing 3,4-DHPEA and *p*-HPEA residuals (Table 2) [18]. More recently, Rovellini and co-workers [14] identified flavonoids (such as luteolin and apigenin) in EVOO. Another class of chemical compounds found in EVOO are lignans, which include (+)-1-acetoxypinoresinol and (+)-1-pinoresinol (Table 2) [19,20]. These chemical compounds (mainly found in the olive pulp and in the woody portion of the seed) are released in EVOO during the extraction process. Table 2 shows the average concentrations of EVOO (+)-1-acetoxypinoresinol and (+)-1-pinoresinol. A detailed analysis of these values singled out that their concentration in EVOO varies less compared to the concentration of secoiridoids, due to the fact that the extent to which they are found in EVOO depends principally on the agronomic conditions required to cultivate olive trees. On the other hand, the technological parameters adopted during the oil extraction process impact slightly on the concentration of EVOO lignans [5]. A quantitative evaluation on several individual hydrophilic phenols of EVOO was performed by HPLC and the averaged concentrations, expressed as median, of prevalent secoiridoids, phenolic acid and phenolic alcohols of EVOO are reported in Table 2.

**Table 1:** Phenolic compounds in Extra Virgin Olive Oil (EVOO).

Phenolic acids and derivatives	Phenolic alcohols	
Vanillic acid	(3,4-Dihdroxyphenyl) ethanol (3,4 DHPEA)	
Syringic acid	(*p*-Hydroxyphenil) ethanol (*p*-HPEA)	
*p*-Coumaric acid	(3,4-Diidrossifenil)etanolo-glucoside	
*o*-Coumaric acid		
Gallic acid	**Lignans**	**Flavones**
Caffeic acid	(+)-1-Acetoxypinoresinol	Apigenin
Protocatechuic acid	(+)-Pinoresinol	Luteolin
*p*-Hydroxybenzoic acid		
Ferulic acid	**Hydroxy-isocromans **	
Cinnamic acid		
4-(acetoxyethil)-1,2-Dihydroxybenzene		
Benzoic acid		
Secoiridoids		
Dialdehydic form of decarboxymethyl elenolic acid linked to 3,4-DHPEA (3,4 DHPEA-EDA)		
Dialdehydic form of decarboxymethyl etenolic acid linked to *p*-HPEA (*p*-HPEA-EDA)		
Oleuropein aglycon (3,4 DHPEA-EA)		
Ligstroside aglycon		
Oleuropein		
*p*-HPEA-derivative		
Dialdehydic form of oleuropein aglycon		
Dialdehydic form of ligstroside aglycon		

**Table 2:** Chemical structures and average values (mg/kg) of the prevalent phenolic alcohols, phenolic acids and secoiridoids of EVOO calculated using 210 oil samples obtained in industrial plants ^a^ [4].

Class	Compounds	Chemical structure	Concentration		
			*Median*	*Lower quintile*	*Upper quintile*
*Phenolic acids*	Vanillic acid	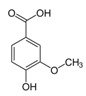	0,2	0	0,3
	Caffeic acid	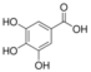	0,4	0,2	0,7
*Phenolic alcohols*	(3,4-Dihdroxyphenyl) ethanol (3,4 DHPEA)	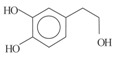	1,8	1	3,6
	(*p*-Hydroxyphenyl) ethanol (*p*-HPEA)	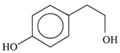	1,9	0,6	5
*Secoiridoids*	Dialdehydic form of decarboxymethyl elenolic acid linked to 3,4-DHPEA (3,4 DHPEA-EDA)	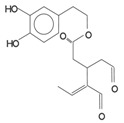	185,7	48,2	631,1
	Dialdehydic form of decarboxymethyl elenolic acid linked to *p*-HPEA (*p*-HPEA-EDA)	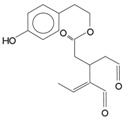	36,1	22,5	78,8
	Oleuropein aglycon (3,4 DHPEA-EA)	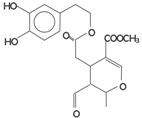	126,3	61	231
	Ligstroside aglycon (*p*-HPEA-EA)	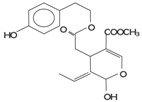	n.d.	n.d.	n.d.
*Lignans*	(+)-1-Acetoxypinoresinol	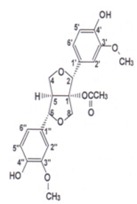	24,6	12,9	30,8
	(+)-1-Pinoresinol	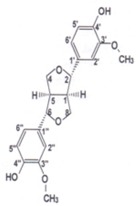	14,4	8,8	47,7

^a^ Unpublished results. The concentration of hydrophilic phenols was evaluated by HPLC as previously reported by Montedoro *et al*. [16].

As we pointed out before, secoiridoids represent the larger fraction of hydrophilic phenols in EVOO. In particular, their chemical structure is represented by the dialdehydic form of decarboxymethyl elenolic acid linked to 3,4-DHPEA or *p*-HPEA (3,4-DHPEA-EDA or *p*-HPEA-EDA), an isomer of oleuropein aglycon (3,4-DHPEA-EA) and ligstroside aglycon (*p*-HPEA-EA). Montedoro *et al*. [16,21,22] were the first to identify secoiridoids in olive oil and in 1993, they also associated their related chemical structure. This was further confirmed by other authors [19,23] (Table 1). These compounds (aglycon derivatives of secoiridoid glucosides contained in the olive fruit) are generated during the mechanical extraction process of the oil, by means of the reactions of oleuropein, demethyloleuropein and ligstroside hydrolysis, catalysed by endogenous β-glucosidases [24]. In fact, olives contain a large amount of phenolic compounds, the concentration of which ranges between 1% and 3% of the weight of the fresh pulp [25]. The aforementioned precursor compounds, in particular, are the most abundant secoiridoid glucosides in the olive fruit. On the contrary, hydroxy-isochromans and oleuropein glucoside (with a range of 5–60 µg/kg) are considered as minor hydrophilic phenols of EVOO.

The concentration of phenols in EVOO is strongly affected by agronomic factors, such as the area of origin, the cultivar, the stage of fruit ripening and also by several agronomic procedures and the technological, operative conditions of the oil extraction process [4,5,10,11,26,27,28,29,30,31,32,33,34,35,36,37,38]. As regards the technological aspects, the processing parameters, such as the operative conditions of crushing and malaxation and of the extraction system, are of great importance to determine the oil hydrophilic phenol content [4,5,34,35,36,37]. In many EVOOs, phenolic compounds are usually present in various concentrations, ranging from 50 to 940 mg/kg.

In this perspective, studies involving humans and animals (*in vivo* and *in vitro*) have demonstrated that olive oil phenolic compounds have potentially beneficial, biological effects resulting from their antioxidant, antimicrobial and anti-inflammatory activities. This review summarizes the current knowledge of the biological properties and the characteristic pungent and bitter taste of olive oil phenolic compounds. Accordingly, the paper is divided into the following sections: Section 2 discusses the antioxidant activities of phenolic compounds; Section 3 illustrates the effects of hydrophilic phenols on health, and Section 4 considers some of the sensorial aspects.

## 2. Antioxidant Activities of Hydrophilic Phenols of EVOO

**Figure 1:**
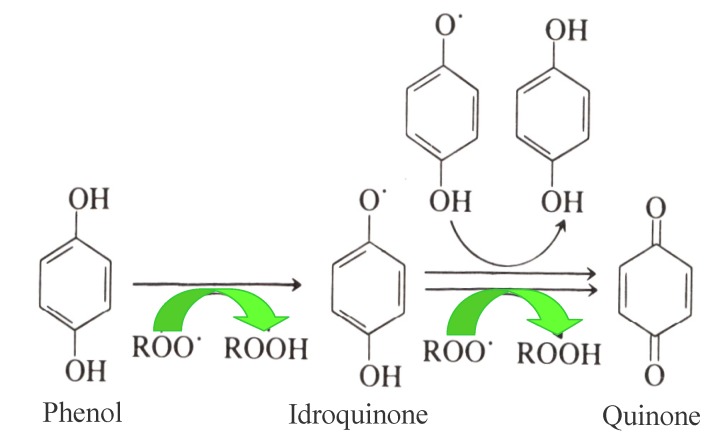
Mechanism of the antioxidant activity of Olive Phenols (Ops) on EVOO.

Antioxidants, as already mentioned, play a key role in the shelf life of EVOO due to their biological activity delaying oxidation processes. In this respect, the primary antioxidants inhibiting oxidation processes in EVOO are OP, which act as chain breakers by donating radical hydrogen to alkylperoxyl radicals, produced by lipid oxidation and the formation of stable derivatives during the reaction (Figure 1). Simple phenols, lignans and secoiridoids represent the phenolic fraction of EVOO, and both their antioxidant properties and their antioxidant activities have been extensively studied in the past [4,5]. Most of these studies focused on the relationships between OP and the relative EVOO shelf-life, using the AOM (Active Oxygen Method) and Rancimat [20,39,40] experimental accelerated methods to investigate them. Moreover, the correlation between total phenols, their antioxidant activity and EVOO shelf life (assessed using a colorimetric method on the methanolic extract of EVOO, the ORAC (Oxygen Radical Absorbance Capacity) test and the Rancimat method, respectively) was confirmed very recently [5,41,42].

However, the same high antioxidant power of 3,4-DHPEA was also shown in an evaluation of the antioxidant activity of some specific hydrophilic phenols (3,4-DHPEA, *p*-HPEA and phenolic acids, such as caffeic acid, *p*-coumaric acid, ferulic acid, syringic acid and vanillic acid) in refined olive oil and in sunflower oil, [4]. Baldioli *et al*. [39] also investigated this property, with a specific study of the oxidative stability of EVOO in several secoiridoids, which were isolated from EVOO using the Rancimat test. They tested, the resistance to oxidation of some oil samples at a temperature of 120 °C, by exposing them to a flow of dry air. The results obtained indicated that *o*-diphenols (3,4-DHPEA, 3,4-DHPEA-EDA and 3,4-DHPEA-EA) showed a higher antioxidant activity than *p*-HPEA and α-tocopherol [39]. These results also confirmed the fact that the natural antioxidants of EVOO with the highest antioxidant efficacy were 3,4-DHPEA and secoiridoids (in particular 3,4-DHPEA-EDA and 3,4-DHPEA-EA previously mentioned) characterized by a molecular structure containing 3,4-DHPEA, and that the antioxidant activity depends on the relative concentrations of these phenols in EVOO.

Artajo *et al*. [43] also used the Rancimat test in order to study the antioxidant power of secoiridoids. They discovered that the strongest antioxidant effects were observed when 3,4-dihydroxy and 3,4,5-trihydroxy residuals are linked to an aromatic ring, such as oleuropein, 3,4-DHPEA-EDA, and the methylated form of 3,4-DHPEA-EA.

Furthermore, Carrasco-Pancorbo *et al*. [44] assessed the antioxidant activity of different EVOO polyphenols. In order to carry out the evaluation, they used the DPPH (1,1,-diphenyl-2-picrylhydrazyl) method, the OSI (Oxidative Stability Instrument) and the flow injection analysis (FIA)—amperometry and the electrochemical method in cyclic voltammetry. Thus, they confirmed the possibility that phenols act as hydrogen donors and also demonstrated the fact that oxidation in EVOO is inhibited by an increase in the number of hydroxyl groups in the structure of OPs. In particular, they were able to demonstrate that compounds linked with a *o*-dihydroxyl functionality had a high antioxidant activity, due to the formation of intramolecular hydrogen bonds observed during the reaction with free radicals. Moreover, the phenol O–H bond is weakened by electron-donating substituents in the “ortho” position and they also stabilize the phenoxyl radical. Therefore, the outcomes of the three aforementioned tests seem to confirm the importance of the hydroxyl groups, which enhance antioxidant activity, as in hydroxytyrosol (3,4-DHPEA), 3,4-DHPEA-EDA, and 3,4-DHPEA-EA, which were found to be the strongest antioxidants. On the other hand, the –COOCH3 fragment, such as in oleuropein aglycon, gives rise to a decrease in the antioxidant capacity due to its inability to act as electron donor [44].

The antioxidant power of lignans was recently studied by Owen *et al*. [19]. They correlate the radical scavenging ability of the EVOO phenolic extract with a concentration of lignans, although other authors did not recognize the observed antioxidant activity of these phenolic compounds [41,43,44].

In 2007, hydroxytyrosyl acyclodihydroelenolate and *p*-coumaroyl-6-secologanoside (comselogoside) biophenolic secoiridoids were isolated, purified and structurally identified by Obied *et al*. [45], in Australian Frantoio olive mill waste (OMW) extracts. Another important feature observed in both biophenolic secoiridoids was that their *in vitro* DPPH scavenging ability is similar to that of 3,4-DHPEA and oleuropein [46]. In particular, hydroxytyrosol acylclodihydroelenolate was revealed to be more effective in the radical scavenging process than 3,4-DHPEA and oleuropein, due to its linear structure making the sterically hindered DPPH radical more accessible. More recently, Angelino *et al*. [42] assessed the composition of the phenolic fraction and the antioxidant capacity (which was estimated using the ORAC method) of oil samples, as well as of the related OMW obtained from two different ripening stages (early and advanced) of the Leccino olive cultivar, in order to compare them and to discover how the phenolic fraction was distributed among the two phases (oil and water) under examination. This work assessed the antioxidant activity of the single phenolic compounds (3,4-DHPEA, *p*-HPEA and 3,4-DHPEA-EDA) contained in the OMW purified extract (OMW-pe) by using chemical (ORAC) and cellular (CAA-RBC) methods. They found that the antioxidant activity of 3,4-DHPEA is higher than that of the other OPs, whereas the estimated antioxidant activity when considering OMW-pe is similar to that of 3,4-DHPEA-EDA. On the other hand, the CAA-RBC results obtained for OMW-pe and the three phenolic compounds under examination indicated that they are able to permeate through the cell membrane. Moreover, the values obtained from the ORAC analysis were confirmed by the CAA-RBC results and, in particular, it was discovered that 3,4-DHPEA exhibits a greater capacity for cellular antioxidant protection compared to *p*-HPEA; finally, they recorded a lower value for OMW-pe, similar to that of 3,4-DHPEA-EDA.

Very recently, several simulations of the behavior of EVOO during frying and other cooking processes have been carried out [5,47,48,49]. The most interesting results of these simulations point to the fact that EVOO is extremely stable during deep frying of food (e.g., potatoes), especially at high temperatures [50,51,52] and in the microwave; this seems to indicate that the consumption of OPs contributes to preserving EVOO from oxidative processes, due to its fast reaction with lipid radicals. In fact, the OPs with a higher antioxidant potential (such as 3,4-DHPEA, 3,4-DHPEA-EDA and 3,4-DHPEA-EA) sharply decrease during heating.

In this respect, Gomez-Alonso *et al*. [53] studied the decrease in the concentration of OPs during several frying repetitions extensively. The results indicated that the amount of OPs was decreased by 40%–50% of the original concentration after 10 min at a temperature of 180 °C (first process). The remaining polyphenolic fraction decreased by less than 10% after six frying operations.

On the other hand, both *p*-HPEA and the ligstroside derivatives, such as *p*-HPEA-EDA and *p*-HPEA-EA, proved highly stable during frying simulation and microwave cooking, providing the confirmation that they are unsuitable for preventing an oxidation reaction in EVOO during cooking processes [54,55]. The stability of tyrosol and its secoiridoid derivatives was further confirmed by other authors: they observed a smaller reduction in their concentration during 12 frying processes than in the hydroxytyrosol family [53].

Moreover, other authors measured the resistance to thermal treatments not only of some secoiridoids derivatives, such as 3,4-DHPEA, elenolic acid, 3,4-DHPEA-EDA and 3,4-DHPEA-EA, but also of lignans. The outcomes of the comparison indicated that lignans are the most thermal resistant compounds in the comparison and that the secoiridoids under examination deteriorated with the thermal treatment faster than other phenols of EVOO, such as 3,4-DHPEA acetate and *p*-HPEA-EA [55].

Pellegrini *et al*. [56] found that α-tocopherol (vitamin E) was stabilized by EVOO polyphenols during the heating of olive oil, determining the nutritional value of the cooked foods. This was due to the combined action of phenolic compounds and vitamin E in inhibiting the oxidation processes and in providing a balanced protection under thermal stress.

Over the last decade, many efforts have been spent on identifying the mechanism of the formation of acrylamide whilst frying amylaceous products. In fact, this compound has been classified as carcinogen and must, therefore, be monitored. However, its formation mechanism during cooking at high temperature is not completely clear, but from recent studies, it appears to be related to the Maillard reaction, involving amino acids and reducing sugars. In particular, they believed asparagine, the main amino acid in potatoes, to be its precursor [57,58]. The acrylamide content evaluated in fried potatoes appears to be positively correlated to the colour of the fried food [58].

Napolitano *et al*. [59] proposed to enrich EVOO with ortho-diphenolic compounds for the purpose of decreasing the acrylamide formation in mild and moderate frying conditions. In fact, these polar antioxidants contribute to inhibiting oxidation in the lipophilic media by acting at the interface between the oil and the polar phase, whereas acrylamide is produced during potato frying. Therefore, it can be affirmed that frying with oil with a high polar compound content is effective in reducing the formation of acrylamide, thanks to the conservation of a chemical reducing environment. Thus, the formation of acrylamide was delayed when dihydroxyphenolic compound enriched EVOO was used.

## 3. Healthy Aspects of Hydrophilic Phenols of EVOO

**Figure 2:**
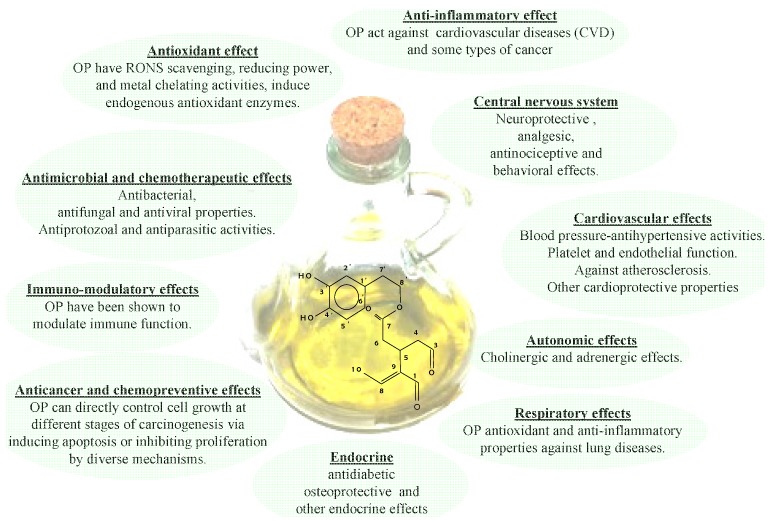
Healthy properties of Ops.

As mentioned previously, the efforts spent in recent years studying EVOO phenolic compounds can be ascribed to the fact that these substances show many healthy benefits, including the reduction of the risk factors of coronary heart disease, the prevention of several chronic diseases (for example, atherosclerosis), cancer, chronic inflammation, strokes and other degenerative diseases [60,61,62,63,64,65,66,67,68,69,70], as depicted in Figure 2. In that Figure a schematic representation of the correlation among EVOO health properties and phenolic compounds [7], 3,4DHPEA-EDA in particular [15], was given. In particular, it appears there are different, interconnected mechanisms, as a result of which OPs bear these healthy effects. In fact, OPs work inside cellular compartments as the first line of defense against free radicals, thanks to their antioxidant capacity, such as cellular redox status modulation by enzyme systems. Moreover, the formation and removal of Reactive Oxygen Species (ROS) affects the oxidation processes inside cells (*i.e.*, oxidative stress). In this respect, a large number of acute and chronic, degenerative diseases probably depend on unbalanced levels of ROS, which drive the oxidation process of Low Density Lipoproteins (LDL). In particular, this oxidation process represents one of the first stages of the onset of atherosclerotic lesions [71] and it is also believed to be responsible for DNA modifications, which, as is well known, give rise to the carcinogenic process [72]. In this respect, a reduced activity of the LDL oxidation process, probably due to diets containing olive oils with a high OP content, was observed by several authors, both *in vitro* and *in* *vivo*. Therefore, the efficacy of OPs was made clear not only by considering their capacity as antioxidants, but also by considering hypercholesterolaemic effects. In particular, extensive studies have been carried out on the consumption of EVOO, which guarantees the intake of the OPs supposedly playing a key role in human health. Moreover, an important claim by the EFSA (European Food Safety Authority) was released in 2011 [8], based on several scientific investigations concerning the role of phenols in human health and indeed, the effectiveness of the ingestion of OPs (5 mg/day) on protecting LDL from oxidation.

A very important study recently carried out by Castañer *et al*. [73] focused on assessing the ability of OPs to modulate the human *in vivo* expressions of atherosclerosis-related genes, in which the LDL oxidation process plays an important role. The outcomes of the experiment seem to clearly indicate that an intake of phenol enriched EVOO is effective in reducing LDL oxidation, due to the increased antioxidant content of the LDL particle [74,75].

In 2010, two hundred, healthy, non-smoking males from six centers of five European Countries were involved in the EUROLIVE study. They were divided into three groups, each of which was fed 25 mL/day of raw EVOO with a high (366 mg/kg), medium (164 mg/kg) and low (3 mg/kg) phenolic content, respectively, in a randomized, cross-over, double-blind and controlled trial, with a Latin square for three treatments in the cross-over randomized trial. A two-week washout period was used before each EVOO intervention, whereas the intervention period lasted three weeks. They observed a decrease in lipid oxidative damage, in addition to an increase in the cholesterol level of High Density Lipoproteins (HDL), which are strongly dependent on the phenolic content of EVOO. Moreover, the increase in the dependent HDL level of OPs has been observed in other studies on humans [76,77,78,79]. In fact, the increase in HDL cholesterol levels is one of the goals of current, cardiovascular disease therapies [76].

The relationships between the positive effects on health due to OPs and maintaining normal blood HDL cholesterol levels were evaluated by the EFSA. However, there is a need for further experimental findings in order to assess this cause-and-effect relationship [80].

The fact that the functionality of the HDL should be more important than its amount in the blood has been the subject of recent studies. HDL functionality could possibly be related to the promotion of cholesterol efflux from macrophages in the so-called “reverse cholesterol transport” process [81]. It has also been observed that this functionality is affected by HDL oxidation [82,83], which, on the other hand, could be counterbalanced by the antioxidant activity of OPs.

Moreover, feeding 400 mg/kg of EVOO OPs has been proved to improve endothelial function in hypercholesterolemic patients in the postprandial state [84], whereas the daily administration of 340 mg/kg of EVOO OPs for 4 months improved the endothelial function in patients with early atherosclerosis [85].

An improvement of the endothelial function was also observed by Moreno-Luna *et al*., who carried out a trial on 24 young women with high-normal blood pressure or stage 1 essential hypertension [86]. In that case, they were given 30 mg/day of OP-enriched EVOO as well as OP-free EVOO in a double-blind, randomized, crossover dietary intervention study. They observed a decrease in blood pressure in particular.

The healthy effects of hydroxytyrosol [87] and its glucuronidated metabolites [88] were identified by recent *in vitro* investigations. These studies pointed out the capacity of both the aforementioned species to inhibit oxidative damage caused by various ROS, as in the case of H_2_O_2_, which is recognized as a major cause of endothelial dysfunction [89].

Moreover, platelet aggregation, a key factor in the formation of thrombus and myocardial infarction or angina, has been proved to be affected by OPs. In fact, the activity of the EVOO phenol DHPE (2-(3,4-di-hydroxyphenyl)-ethanol) seems to interfere with 400 mM platelet aggregation *in vitro* [88]. In this regard, some phenolic compounds appear to have strong, anti-inflammatory effects, both *in vitro* and *in vivo* [90,91,92,93,94]. In fact, oleocanthal, which Servili *et al*. [95] identified for the first time in olives as the dialdehydic form of deacetoxy-ligstroside aglycon (*p*-HPEA-EDA), shows some features which recall the pharmacological effects of ibuprofen, a modulator of inflammation and analgesia [96]. It was also observed that the activity of both enantiomers of *p*-HPEA-EDA is responsible for the dose-dependent inhibition of COX-1 and COX-2 (which are cyclo-oxygenase enzymes, catalyzing key-steps in the biochemical inflammation pathways derived from arachidonic acid) activities that can be correlated to the action of ibuprofen. Indeed, the authors suggested the likelihood of a decreased risk in developing certain cancers and lower platelet aggregation in the blood, thanks to a prolonged consumption of oleocanthal, as in the case of its ibuprofen-like, COX-inhibiting activity.

In the last decade, several studies carried out *in vitro* and *in vivo* have assessed the antiproliferative and pro-apoptotic effects on cancer cells due to the antioxidant activity of OP, obtained from olive oil and from the by-products of mechanical extraction [97,98,99]. In a recent work, Fabiani *et al*. [100] stressed the fact that the different phenolic composition of EVOO extracts reflects different chemopreventive activities *in vitro* towards HL60 cellular lines. The various chemopreventive effects shown by the extracts seem to depend directly on the phenolic composition rather than on their total amount. In fact, the antiproliferative and pro-apoptotic effects are due to 3,4-DHPEA-EDA and *p*-HPEA-EDA, which are the main components of EVOO extracts. It is important to point out that the concentrations of 3,4-DHPEA-EDA and *p*-HPEA-EDA in the phenolic extract (with a concentration of 5 µg/mL) used in the culture medium were lower than the pure 3,4-DHPEA-EDA and *p*-HPEA-EDA concentrations (IC_50%_ 30–35 and 7–8 μM, respectively) needed to obtain a significant effect. Therefore, some synergies between different phenols are likely to be established within the extract.

The negative correlation of 3,4-DHPEA-EDA and *p*-HPEA-EDA with the antiproliferative and pro-apoptotic effects can be explained in terms of their concentration. In fact, the concentrations of 3,4-DHPEA-EDA and *p*-HPEA-EDA, when the phenolic extract is dissolved into the culture medium at a concentration of 5 µg/mL, were found to be lower than those needed to achieve an effect on HL60 cells (>10 µM for 3,4-DHPEA and >250 µM for *p*-HPEA).

As regards lignans, pinoresinol appears to have an insignificant pro-apoptotic activity which, in any case, is sufficient to inhibit the proliferation of HL60 cells, when its concentration is of the order of 10–100 µM, even though its concentration in the phenolic extract was lower. On the other hand, although several studies have demonstrated the chemopreventive properties of 3,4-DHPEA in terms of its antiproliferative and pro-apoptotic effects, the effects of EVOO phenolic extracts on cancer cells are still unclear.

Phenolic compounds have recently been associated with the likelihood of preventing the risk of Alzheimer’s disease and of reducing its related effects, although the mechanism by which the EVOO phenols exert their neuroprotective effects is not completely clear [101]. Alzheimer’s disease is a neurodegenerative disease characterized by an accumulation of amyloid plaques and neurofibrillary tangles in the brain [102]. Recently, several mechanisms have been proposed to describe the role that hydrophilic phenols play in the reduced incidence of Alzheimer’s disease [103,104,105]. Farr *et al*. [106] found that EVOO OPs (210 mg/kg) have healthy effects on learning and memory deficits of ageing and diseases, such as those related to the overproduction of the amyloid-β peptide. In this study, they demonstrated how OPs were able to reverse oxidative damage in the brains of mice, an age-related learning/memory impairment model associated to an increased amyloid-β protein production and brain oxidative damage. This effect was enhanced by increasing the EVOO OP concentrations (from 210 to 1050 mg/kg).

Abuznait and co-workers [107] carried out experiments *in vitro* and *in vivo* on the healthy effects of EVOO hydrophilic phenolic compounds on Alzheimer’s disease. They demonstrated that these effects could be related to the ability of EVOO OPs to reduce the accumulation of amyloid plaques and to enhance β-Amyloid clearance from the blood-brain barrier, thanks to the fact that EVOO OPs improve the action carried out by the two major transport proteins, P-glycoprotein (P-gp) and the LDL lipoprotein receptor-related protein 1 (LRP1) [107].

Finally, as regards the anti-microbial properties of phenolic compounds, it has been observed that the administration of EVOO phenolic compounds also affects the gut microbial balance, due to the fact that they are not completely absorbed into the upper parts of the gastrointestinal tract, whereas they are metabolized in the lower parts by the gut microflora [108]. It is well known that inflammatory signaling pathways are modulated by gut pathogens [109]. Therefore, phenolic compounds could be used to contrast the development of atherosclerosis, thanks to their anti-microbial activity. Moreover, the growth of some beneficial bacteria (such as *Lactobacillus*) can be selectively enhanced by the OPs [110].

## 4. Sensorial Aspects of Hydrophilic Phenols of EVOO

The OPs of EVOO strongly affect its sensory properties. The fact that the EVOO phenolic fraction has a strong impact on bitterness, astringency and pungency has been the subject of several past studies [4,5,111,112,113,114]. In this respect, tyrosol, hydroxytyrosol and their relative derivatives are considered responsible for “bitter” EVOO.

In order to establish the correlation between the bitterness of EVOO and the related chemical compounds behind it, Garcia *et al*. [115] sensorially measured the bitterness of EVOO. By assessing the overall amount of the two secoiridoids derivatives of hydroxytyrosol, the dialdehydic form of decarboxymethyl oleuropein aglycon and the aldehydic form of oleuropein aglycon, they also estimated it chemically. In fact, the sum of the contents of the previously mentioned secoiridoids represents an objective estimation of the oil taste sensation. They found a decrease in the bitterness of EVOO, clearly due to the temperature value adopted during the extraction process. The sensory attributes, including bitterness, were the subject of a quantitative, descriptive analysis. They were estimated, thanks to an analytical panel using a structured, six-point scale, as illustrated in Table 3. A particularly good correlation was discovered between the overall content of the two secoiridoids under examination and the bitterness of EVOO for each olive variety investigated. 

Over the last decades, the fact that the attributes of the taste of EVOO had to be ascribed either to the total phenolic content of EVOO or to the lignans fraction, has been the subject of debate. Moreover, a statistical correlation between the total amount of secoiridoids and EVOO sensory responses, such as “astringency” and “bitterness”, has been the subject of a certain number of studies. However, very little data identifying the chemical structures of secoiridoids and their taste attributes has been published.

**Table 3:** Values of the six points adopted by the panel (Note: with permission from [115], Copyright^©^ American Chemical Society, 2001).

Reference value	Sensory attributes
0	absence of attribute
1	simple perception
2	light presence
3	middle presence
4	strong intensity
5	highest intensity

In this regard, many authors have hypothesized that the main contributors to the bitterness of EVOO are those compounds with an aromatic ring in their chemical structure, as in the case of the secoiridoid derivatives of oleuropein (3,4-DHPEA-EDA and 3,4-DHPEA-EA) [116,117]. Furthermore, Tovar *et al*. [117] confirmed this hypothesis by proving that *p*-HPEA-EDA is responsible for the bitter and pungent sensory notes of EVOO.

Moreover, Gutiérrez-Rosales *et al*. [118] isolated every secoiridoid derivative for the first time in 2003, by using the preparative HPLC technique to assess the intensity of its bitterness. The authors associated the relevant peaks with 3,4-DHPEA-EDA, 3,4-DHPEA-EA and *p*-HPEA-EDA, which were mainly responsible for the bitterness of EVOO. In particular, they observed a strong correlation between the content of 3,4-DHPEA-EDA (*r* = 0.9819 *p* ≤ 10^−3^), *p*-HPEA-EDA (*r* = 0.9830 *p* ≤ 10^−3^) and 3,4-DHPEA-EA (*r* = 0.7929 *p* ≤ 10^−2^) and the intensity of the bitterness.

A recent work by Andrewes *et al*. [119] correlated the chemical structures of secoiridoids, such as *p*-HPEA-EDA and 3,4-DHPEA-EDA, with the burning/pungent sensory notes of EVOO. These secoiridoids were isolated in EVOO in two different fractions; one containing *p*-HPEA-EDA featured a strong burning/pungent sensation, whereas the fraction containing 3,4-DHPEA-EDA had a slight burning/pungent sensation, which was perceived more on the tongue. This confirmed the fact that the burning-pungent sensory note in EVOO is mainly caused by *p*-HPEA-EDA. On the other hand, they did not identify any other polyphenolic fractions producing such an intense sensation.

In 2005, Beauchamp *et al*. [96] confirmed the outcomes of the aforementioned study. In fact, they used a synthesized *p*-HPEA-EDA (renamed oleocanthal), dissolved into non-irritating corn oil, and tested the throat-irritant properties of this compound. They found a dose-dependent effect similar to that of the same compound found in EVOO, probably due to the two dialdehydic groups found in the chemical structure.

Recent investigations on *p*-HPEA-EDA have been carried out in order to characterize the spatial location of irritation it produces. Peyrot des Gachons *et al*. [120] reported that all the regions in the oral cavity are aggravated by irritating, pungent substances, which do not act on one localized area. This implies that a sensory receptor specific to *p*-HPEA-EDA exists in the oropharyngeal region of the oral cavity. More recent studies by Hayes *et al*. [121] identified TRPA1 as the *p*-HPEA-EDA receptor located, from an anatomical point of view, in the oropharyngeal region of the oral cavity [120,122].

## 5. Conclusions

The EVOO quality is intimately affected by its content in phenolic compounds. In fact, the hydrophilic phenols influence not only its shelf-life but also its health and sensory proprieties.

The review has been focused on the evaluation of the antioxidant effects of OPs. In particular, the antioxidant activities and healthy properties of secoiridoids derivatives, such as 3,4-DHPEA, 3,4-DHPEA-EDA, 3,4-DHPEA-EA, *p*-HPEA, *p*-HPEAEDA and lignans, have been taken into account. It was found that the high resistance to oxidation of EVOO is due to oleuropein and 3,4-DHPEA-EA derivatives, while lignans play a secondary role. Moreover, with respect to the healthy properties, these substances show a high antioxidant activity and play a key role in the prevention and/or reduction of chronic degenerative events based on inflammatory processes and chronic-degenerative diseases, such as cardiovascular-cerebral diseases and cancer. Furthermore, it was also found that the sensory notes of EVOO are affected by OPs. In fact, it was demonstrated that open ring *p*-HPEA-EDA is responsible for the strong “pungent” attribute, while closed ring 3,4-DHPEA-EA and *p*-HPEA-EA represent the impact components for the “bitter” note. The 3,4-DHPEA-EDA, which contributes to the sensation of bitter however, plays a marginal role for the “pungent” note.

In recent years, the innovation process in the field of EVOO is being orienting towards a new concept of quality, strictly related to OPs content (which, more than other compounds, are affected by technological processes) to end of producing EVOOs characterized by a strong sensory impact and healthy benefits.
